# Killing of myeloid APCs via HLA class I, CD2 and CD226 defines a novel mechanism of suppression by human Tr1 cells

**DOI:** 10.1002/eji.201041120

**Published:** 2011-04-06

**Authors:** Chiara F Magnani, Giada Alberigo, Rosa Bacchetta, Giorgia Serafini, Marco Andreani, Maria Grazia Roncarolo, Silvia Gregori

**Affiliations:** 1 San Raffaele Telethon Institute for Gene Therapy (HSR-TIGET), Division of Regenerative Medicine, Stem Cells and Gene Therapy, San Raffaele Scientific InstituteMilan, Italy; 2 Mediterranean Institute of Hematology (IME Foundation)Policlinico di Tor Vergata, Rome, Italy; 3 Vita-Salute San Raffaele UniversityMilan, Italy

**Keywords:** Cytotoxicity, Granzyme B, Immune regulation, Type 1 regulatory T cells

## Abstract

IL-10-producing CD4^+^ type 1 regulatory T (Tr1) cells, defined based on their ability to produce high levels of IL-10 in the absence of IL-4, are major players in the induction and maintenance of peripheral tolerance. Tr1 cells inhibit T-cell responses mainly via cytokine-dependent mechanisms. The cellular and molecular mechanisms underlying the suppression of APC by Tr1 cells are still not completely elucidated. Here, we defined that Tr1 cells specifically lyse myeloid APC through a granzyme B (GZB)- and perforin (PRF)-dependent mechanism that requires HLA class I recognition, CD54/lymphocyte function-associated antigen (LFA)-1 adhesion, and activation via killer cell Ig-like receptors (KIRs) and CD2. Notably, interaction between CD226 on Tr1 cells and their ligands on myeloid cells, leading to Tr1-cell activation, is necessary for defining Tr1-cell target specificity. We also showed that high frequency of GZB-expressing CD4^+^ T cells is detected in tolerant patients and correlates with elevated occurrence of IL-10-producing CD4^+^ T cells. In conclusion, the modulatory activities of Tr1 cells are not only due to suppressive cytokines but also to specific cell-to-cell interactions that lead to selective killing of myeloid cells and possibly bystander suppression.

## Introduction

CD4^+^ type 1 regulatory T (Tr1) cells are adaptive IL-10-producing Tregs fundamental in controlling immune responses and in inducing peripheral tolerance both in humans and mice 1, 2. The first indication that Tr1 cells mediate peripheral tolerance in vivo came from SCID patients who developed long-term tolerance to stem cell allograft 1. After that, Tr1 cells have been found to be induced in a variety of in vivo settings 3. Tr1 cells have been recently associated with the induction of persistent mixed chimerism (PMC) in β-thalassemic (β-thal) patients after HLA identical hematopoietic stem cell transplantation (HSCT) 4.

Tr1 cells are induced in the periphery upon chronic Ag stimulation in the presence of IL-10 derived from tolerogenic APC 3. No specific cell markers for Tr1 cells have been identified so far. Therefore, Tr1 cells can be characterized based on their specific cytokine production profile (IL-10^+^, TGF-β^+^, IL-4^−^, IL-2^low^, and IFN-γ^low^). Tr1 cells are Ag-specific, hypo-responsive, and suppress effector T cells mainly by the release of IL-10 and TGF-β 2. It has been hypothesized that a cell-contact-dependent mechanism cooperates with the release of immunosuppressive cytokines in inhibiting immune responses by Tr1 cells, since the addition of neutralizing antibodies against IL-10R and TGF-β did not completely revert suppression mediated by Tr1 cells 5.

Murine CD25^+^ Treg cells express granzyme B (GZB) 6, 7, and induce apoptosis of T and NK cells 8, 9, indicating that GZB-dependent killing of T cells represents one of the mechanisms responsible for Treg-mediated suppression. In line with these findings, CD25^+^ Tregs isolated from GZB-deficient mice have reduced suppression ability compared to CD25^+^ Tregs from wild type mice 8.

Human naturally occurring Tregs (nTregs) or adaptive IL-10-producing Tregs, depending on the mode of activation/generation, can express both granzyme A (GZA) and GZB 10–12. nTregs express GZA or GZB when activated in the presence of low or high concentrations of IL-2, respectively 10, 11. IL-10-producing Tregs generated in vitro by activating CD4^+^ T cells with anti-CD3 and anti-CD46 mAb express only GZB 10, whereas IL-10-producing Tregs induced by HSV-stimulated human plasmacytoid DCs express both GZA and GZB 13. nTregs activated with CD3/CD28 and IL-10-producing Tregs activated with CD3/CD46 were shown to kill different target cells through the adhesion of CD18 10.

In the present study, we investigated the cellular and molecular mechanisms underneath Tr1-mediated cytotoxicity. Results show that polarized Tr1-cell lines and Tr1-cell clones express and release high levels of GZB in an IL-10-dependent manner, and lyse APC via GZB and perforin (PRF). Lysis mediated by Tr1 cells requires HLA class I recognition, lymphocyte function-associated antigen (LFA)-1-mediated adhesion, and stimulation via CD2 and CD226, and consequently is restricted to myeloid APC that express high levels of the ligands of LFA-1 (CD54), of CD2 (CD58), and of CD226 (CD155). GZB^+^CD4^+^ T cells are detected in the periphery of multiple-transfused β-thal patients and in PMC β-thal patients in whom Tr1 cells are present at high frequency, supporting the hypothesis that GZB is relevant also for the in vivo function of Tr1 cells.

## Results

### Human Tr1 cells express and release high levels of GZB

**Figure 1:**
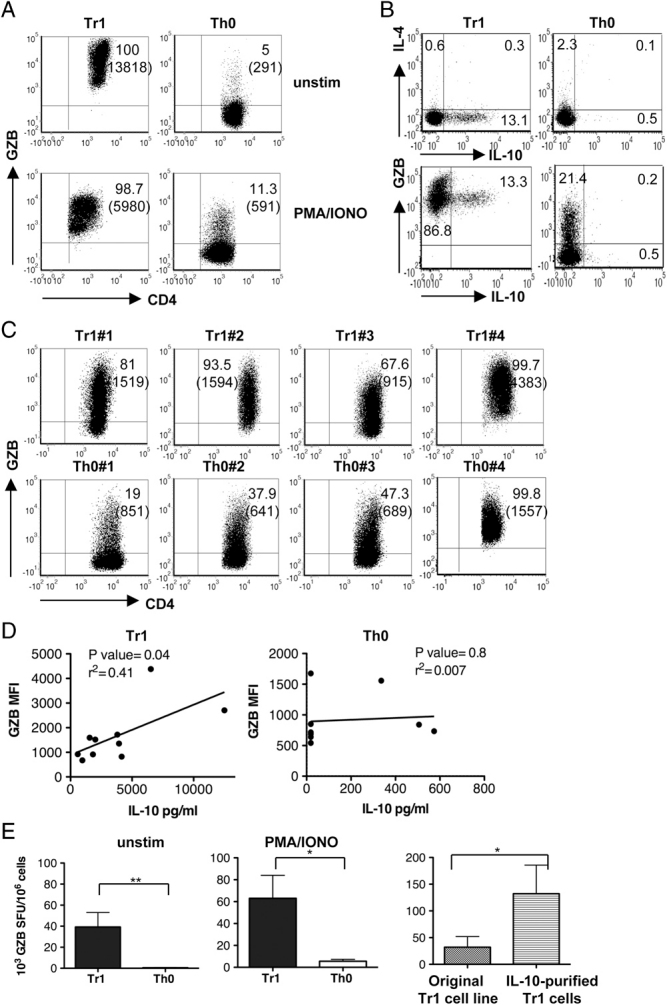
Tr1 cells express and release GZB. (A) GZB expression was determined in Th0- and Tr1-cell lines unstimulated (unstim) or stimulated with PMA (10 ng/mL; Sigma) plus IONO (ionomycin) (150 ng/mL; Sigma) for 6 h. One donor representative of 11 unstimulated donors and of five stimulated donors tested is shown. Numbers represent percentage of positive cells and MFI in bracket. (B) Alternatively, IL-10, IL-4, and GZB expression was determined upon stimulation with Leukocyte Activation Cocktail (BD Pharmingen) for 5 h. One donor out of six donors tested is shown. Numbers represent percentage of positive cells. (C) GZB expression was determined in four unstimulated Tr1- and Th0-cell clones. Numbers represent percentage of positive cells and MFI in bracket. (D) Plot represents IL-10 production expressed as pg/mL versus MFI of GZB expression in each of ten Tr1-cell clones and of nine Th0-cell clones tested. The line represents the linear regression. The *p* value of the correlation and the coefficient of determination (*r*^2^) are reported (two-tailed test). (E) GZB release by unstimulated (unstim) and stimulated with PMA/IONO Tr1- and Th0-cell lines was measured by ELISPOT. The *Y*-axis represents the number of SFU/10^6^ cells. (F) GZB release by original Tr1-cell lines, and purified IL-10-producing cells was measured by ELISPOT. Mean±SE of GZB spots normalized to 10^6^ cells of five (unstim), three (PMA/IONO), and three (F) independent experiments performed in duplicate is shown. SFU, spot forming units ^*^p≤0.05, and ^**^p≤0.005 (one-tailed test).

Tr1 polarized cell lines expressed significantly higher levels of GZB compared to Th0-cell lines (97.3 versus 12.9%, *n*=11, *p*<0.0001, Fig. 1A). Notably, IL-10-producing Tr1 cells represent 10–15% of the polarized population, thus GZB expression is not restricted to this population of cells (Fig. 1B). Tr1-cell lines express also significantly higher levels of GZA compared to Th0-cell lines (58.7% versus 9%, *n*=8, *p*<0.0001, not shown), nevertheless its expression was consistently lower than that of GZB. Tr1-cell lines contained a significantly higher percentage of PRF^+^ cells compared to Th0-cell lines before (8.8 versus 1.8%, *n*=7, *p*=0.015) and after stimulation (13.3 versus 5.1%, *n*=7, *p*=0.007, not shown).

Tr1-cell clones, isolated from peripheral blood of two distinct healthy donors (HD) and defined based on IL-10/IL-4 ratio ≥8 (Table 1), expressed higher levels of GZB compared to Th0-cell clones (Fig. 1C). The MFI of GZB expression was variable among both Tr1- and Th0-cell clones, but it correlated with the amounts of IL-10 produced by Tr1- but not Th0-cell clones (Fig. 1D), suggesting a relationship between the presence of IL-10 in Tr1-cell culture and GZB expression.

**Table 1:** Cytokine profile of T-cell clonesa)

	IL-2 pg/mL	IL-4 pg/mL	IL-10 pg/mL	IFN-γ pg/mL	IL-17 pg/mL
Tr1♯1	48	<9	2015	2205	<30
Tr1♯2	<15	37	1558	7942	<30
Tr1♯3	185	<9	1818	11 015	67
Tr1♯4	<15	728	6528	7093	<30
Tr1♯5	<15	<9	590	766	<30
Tr1♯6	126	97	3808	6187	218
Tr1♯7	<15	22	3941	1796	59
Tr1♯8	<15	<9	4160	52 684	90
Tr1♯9	<15	757	12 500	3723	<30
Tr1♯10	170	127	959	967	<30
Th0♯1	22	>3000	<19	469	<30
Th0♯2	105	1446	<19	1895	<30
Th0♯3	132	2105	<19	3099	<30
Th0♯4	<15	145	336	3124	<30
Th0♯5	294	>3000	<19	2615	<30
Th0♯6	>1000	>3000	574	2567	<30
Th0♯7	527	2523	506	2833	<30
Th0♯8	1211	2597	<19	3099	<30
Th0♯9	362	212	<19	1799	<30

a) Tr1 and Th0 cell clones were stimulated with immobilized anti-CD3 mAb and soluble anti-CD28 mAb. Culture supernatants were collected after 24 h (IL-2) and 48 h (IL-4, IL-10, IFN-γ, and IL-17) and cytokine levels were measured by ELISA.

Ex vivo isolated CD4^+^IL-10^+^ T cells contained a higher percentages of GZB^+^ cells compared to CD4^+^IL-10^−^ T cells (20.6 % versus 2.4%, Supporting Information Fig. 1), indicating that also circulating Tr1 cells express GZB. However, it should be considered that to isolate CD4^+^IL-10^+^ T cells, peripheral blood cells are pre-activated and, as shown in Fig. 1A GZB expression decreased in activated Tr1 cells. Thus, the percentage of GZB observed in ex vivo isolated CD4^+^IL-10^+^ T cells probably does not mirror the in vivo situation.

Tr1-cell lines spontaneously released significantly higher levels of GZB compared to Th0-cell lines (39×10^3^ versus 0.5×10^3^ SFU/10^6^ cells, in Tr1 and Th0 cells, *n*=5, *p*=0.006, Fig. 1E). GZB released by Tr1-cell lines was further increased upon activation (Fig. 1E). Purified IL-10-producing Tr1 cells secreted significantly higher amounts of GZB compared to the original Tr1-cell lines (132×10^3^ versus 32×10^3^ SFU/10^6^ cells, *n*=3, *p*=0.05, Fig. 1F), indicating that IL-10-producing Tr1 cells are the main GZB producers in culture. Notably, GZB expression in purified IL-10-producing Tr1 cells was higher compared to that of non-IL-10-producing T cells contained in polarized Tr1-cell population (data not shown), sustaining the conclusion that GZB expression in non-IL-10-producing T cells present in Tr1-polarized populations is the result of exposure to IL-10-derived from Tr1 cells. Overall, these data demonstrate that Tr1 cells expressed and released high levels of GZB in an IL-10-dependent manner, and that non-IL-10-producing T cells present in the polarized cultures expressed GZB as a result of IL-10 exposure.

### Tr1 cells specifically kill myeloid cells

**Figure 2:**
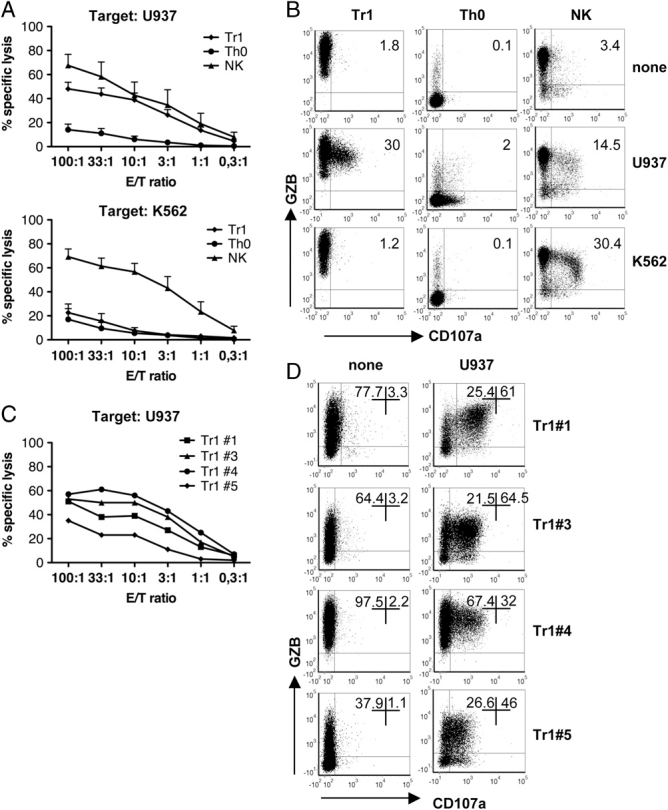
Tr1-cell lines and Tr1-cell clones specifically lyse target cell of monocytic origin. (A) The cytotoxic activity of Tr1- and Th0-cell lines against U937 and K562 target cells was determined by ^51^Cr release. NK cells from the same HD were used as positive controls. Mean±SE of six donors for U937 and of five for K562 performed in duplicate are reported. (B) Tr1- and Th0-cell lines were co-cultured with U937 and K562 target cells at 10:1 E:T ratio. Degranulation in the presence of GZB was measured by co-expression of CD107a and GZB in CD4^+^ T cells. NK cells from the same HD were used as positive controls. One donor representative of ten donors performed in eight independent experiments for U937 and of four donors performed in three independent experiments for K562 is shown. Numbers represent percentage of CD107a^+^GZB^+^ cells. (C) Cytotoxic activity of four Tr1-cell clones against U937 target cell was determined by ^51^Cr release. (D) In parallel, Tr1-cell clones were co-cultured with U937 target cell at 10:1 (E:T) ratio and degranulation in the presence of GZB was measured by co-expression of CD107a and GZB in CD4^+^ T cells. Numbers represent percentage of CD107a^+^GZB^+^ cells.

Tr1-cell lines efficiently lysed U937 cells, a monocytic cell line, but not K562 cells, an erythroleukemic cell line (Fig. 2A), or Daudi, a B lymphoblast cell line, or Jurkat, a T leukemic cell line (Supporting Information Fig. 2). Th0-cell lines exerted limited lytic activity on the cell lines tested (Fig. 2A and Supporting Information Fig. 2).

When Tr1 cells were co-cultured with U937 cells, high percentage of CD107a^+^GZB^+^ cells was observed (on average 26% of CD107a^+^GZB^+^ cells in Tr1 cells cultured with U937 cells compared to 3% in Tr1 cells alone, *p*=0.0002, Fig. 2B), consistent with the lysis assessed by ^51^Cr assay (Fig. 2A). As expected, percentages of CD107a^+^GZB^+^ cells within the Tr1 cells co-cultured with K562, Daudi, or Jurkat cells were low and similar to those observed in Tr1 cells cultured alone (Fig. 2B and not shown). Despite the ability of Th0-cell lines to degranulate when activated with U937 cells, as demonstrated by CD107a staining, they were unable to lyse these target cells. The percentages of CD107a^+^GZB^+^ cells were indeed similar to those of observed in Tr1-cell cultures alone (Fig. 2B).

Four different Tr1-cell clones efficiently lysed U937 but not K562 cells (Fig. 2C and D and not shown). Tr1 clone ♯4, that displayed the highest lytic ability (Fig. 2C), had also the highest GZB expression (99.7%, MFI 4383, Fig. 2D left panel) and secreted the highest level of IL-10 (6528 pg/mL, Table 1), whereas Tr1 clone ♯5 had the lowest lytic ability (Fig. 2C), the lowest GZB expression (39%, MFI 798, Fig. 2D left panel), and secreted the lowest levels of IL-10 (590 pg/mL, Table 1). Tr1 clones ♯1 and ♯3 killed U937 cells similarly (Fig. 2C) and had similar levels of GZB (81% MFI 1519 and 67.6% MFI 915, respectively, Fig. 2D left panel) and IL-10 production (2015 pg/mL and 1818 pg/mL, respectively, Table 1). The percentages of CD107a^+^GZB^+^ cells in Tr1-cell clones were higher (Fig. 2D) compared to Tr1-cell lines (Fig. 2B), consistent with the fact that Tr1-cell clones are a homogeneous population of IL-10-producing T cells. Notably, Th0-cell clones efficiently lysed U937 cells (Supporting Information Fig. 3) but cytotoxicity was independent of the levels of GZB expression and of their ability to secrete IL-10 (Fig. 1D left panel). These data demonstrated that GZB expression by Tr1 cells correlates not only with IL-10 production but also with their lytic activity against myeloid cell line.

**Figure 3:**
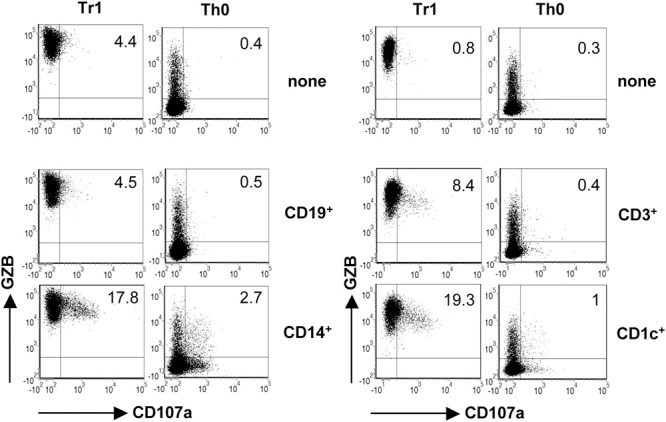
Tr1-cell lines specifically lyse primary monocytes and myeloid DCs. Tr1- and Th0-cell lines were co-cultured with allogeneic freshly isolated CD19^+^, CD14^+^, CD3^+^, and CD1c^+^ cells at 10:1 (E:T) ratio, and degranulation in the presence of GZB was measured by co-expression of CD107a and GZB in CD4^+^ T cells. One donor out of four (CD19^+^), three (CD14^+^), three (CD3^+^), and four (CD1c^+^) donors performed in two independent experiments is shown. Numbers represent percentage of CD107a^+^GZB^+^ cells.

Tr1-cell lines degranulate in the presence of freshly isolated CD14^+^ and CD1c^+^ cells, but not CD19^+^ and CD3^+^ T cells (both allogeneic and autologous, Fig. 3 and Supporting Information Fig. 4A). The mean percentages of CD107a^+^GZB^+^ cells in Tr1-cell lines co-cultured with autologous CD14^+^ cells or CD1c^+^ cells were 27% and 18%, respectively. Similar results were obtained when Tr1-cell lines were co-cultured with allogeneic CD14^+^ cells (21%) and CD1c^+^ cells (18%). Of note, mean percentages of CD107a^+^GZB^+^ cells in Tr1-cell lines co-cultured with autologous or allogeneic CD3^+^ T cells were 7% (*n*=4, Fig. 3) and 6% (*n*=3, Supporting Information Fig. 4A), respectively. Analysis of Annexin V and 7-aminoactinomycin D (7-ADD) staining confirmed that, while CD14^+^ cells are killed, CD3^+^ T cells are not killed when co-cultured with Tr1-cell lines (Supporting Information Fig. 4B).

Overall these findings demonstrate that Tr1-cell lines specifically kill both autologous and allogeneic cells of myeloid origin.

### Tr1-mediated cytotoxicity is dependent on GZB and PRF, and requires HLA class I recognition

**Figure 4:**
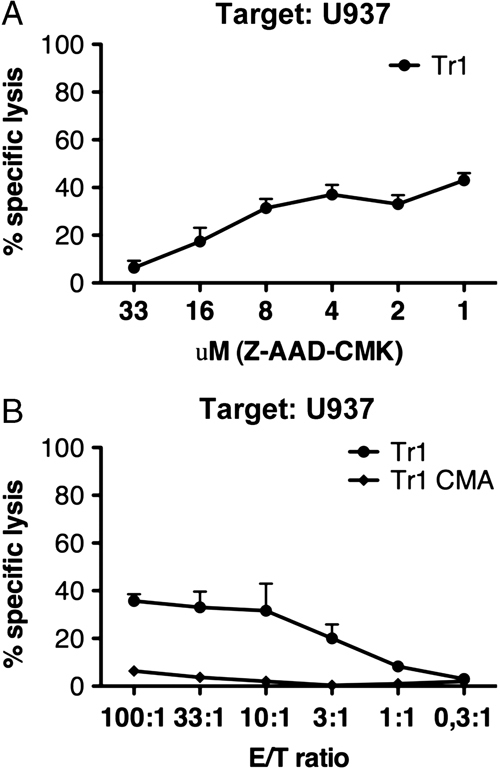
Tr1-mediated cytotoxicity is GZB- and PRF-dependent. Tr1-cell lines were pre-incubated with Z-AAD-CMK at the indicated concentrations (A) or with CMA (100 nM) (B), co-cultured with U937 target cell at 100:1 (E:T) ratio, and cytotoxicity was determined by ^51^Cr release. Mean±SE of three donors are reported.

The role of GZB in the lytic activity mediated by Tr1 cells was demonstrated by the addition of Z-AAD-CMK, an inhibitor of GZB, which completely abrogated the cytotoxic activity of Tr1 cells in a dose-dependent manner (Fig. 4A). Similarly, Tr1-mediated cytotoxicity was nearly abolished when CMA, a PRF inhibitor, was added (Fig. 4B), indicating that both GZB and PRF are required for Tr1-cell-mediated killing.

**Figure 5:**
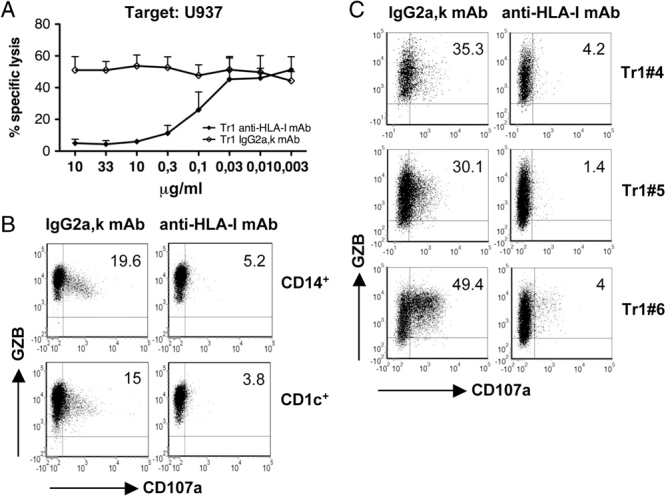
Tr1-mediated cytotoxicity is HLA class I-dependent. (A) Tr1-cell lines were co-cultured with U937 target cell line at 100:1 (E:T) ratio in the presence of anti-HLA class I mAb or IgG2a,k isotype control at the indicated concentrations, and cytotoxicity was determined by ^51^Cr release. Mean±SE of four donors performed in two independent experiments are reported. (B) Tr1-cell lines were co-cultured with freshly isolated autologous CD14^+^ and CD1c^+^ cells at 10:1 (E:T) ratio in the presence of anti-HLA-I mAb or IgG2a,k isotype control, and degranulation in the presence of GZB was measured by co-expression of CD107a and GZB in CD4^+^ T cells. One donor representative of three donors performed in a single experiment is shown. Numbers represent percentage of CD107a^+^GZB^+^ cells. (C) Tr1-cell clones were co-cultured with U937 target cell at 10:1 (E:T) ratio in the presence of anti-HLA-I mAb or IgG2a,k isotype control, and degranulation was measured by co-expression of CD107a and GZB in CD4^+^ T cells. Numbers represent percentage of CD107a^+^GZB^+^ cells.

Addition of a pan anti-HLA-I mAb (clone W6/32) significantly inhibited, in a dose-dependent manner, the killing of U937, CD14^+^, and CD1c^+^ cells (autologous and allogeneic) by Tr1-cell lines and by three distinct Tr1-cell clones (Fig. 5A–C and data not shown). Tr1 cells express a variety of activating killer cell Ig-like receptors (KIRs) including KIR2DS2, KIR2DS3, KIR3DS1, and KIR2DL4, the ligand specific for HLA-G (Table 2). Addition of neutralizing anti-HLA-G mAb (clone 87G) partially inhibited, in a dose-dependent manner, the killing of U937, CD14^+^, and CD1c^+^ cells by both Tr1-cell lines (Supporting Information Fig. 5A and B) and Tr1-cell clones (Supporting Information Fig. 5C), supporting the contribution of stimulatory KIRs in promoting the killing of target cells.

**Table 2:** Tr1-cell lines express activating KIRsa)

	Th0 HD1	Tr1 HD1	Th0 HD2	Tr1 HD2
KIR2DL4	+	+	+	+
KIR2DS1	−	−	+	−
KIR2DS2	+	+	+	+
KIR2DS3	+	+	−	−
KIR2DS4	−	−	−	−
KIR2DS5	−	−	−	−
KIR3DS1	−	−	+	+

a) mRNA expression of human activating KIR genes was assessed in Th0 and Tr1 T-cell lines by KIR typing. Clear visibility (+) or absence (−) of KIR-specific PCR products of two distinct donors tested are shown.

Thus, similar to the NK-mediated killing, Tr1 cells, despite the fact that they are CD4^+^ T cells, require Ag-nonspecific HLA class I recognition and activation via KIRs to lyse target cells.

### Tr1-mediated killing of myeloid cells requires CD54 adhesion and activation via CD2 and CD226

**Figure 6:**
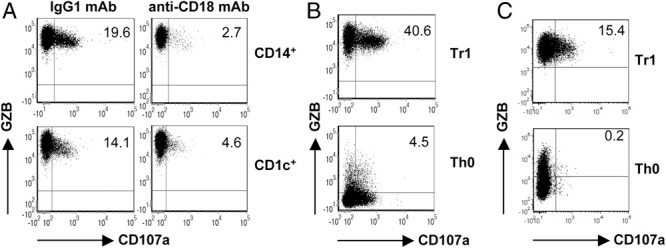
Tr1-mediated cytotoxicity requires CD18 adhesion and activation via CD2 and CD226. (A) Tr1-cell lines were co-cultured with freshly isolated allogeneic CD14^+^ and CD1c^+^ cells at 10:1 (E:T) ratio in the presence of anti-CD18 mAb or IgG1 isotype control, and degranulation in the presence of GZB was measured by co-expression of CD107a and GZB in CD4^+^ T cells. One donor representative of three donors tested in two independent experiments is shown. Numbers represent percentage of CD107a^+^GZB^+^ cells. (B) Tr1- and Th0-cell lines were co-cultured with p815 cells pre-incubated with anti-CD2 mAb at 10:1 (E:T) ratio, and degranulation was determined by co-expression of CD107a and GZB in CD4^+^ T cells. One donor representative of three donors performed in two independent experiments is shown. Numbers represent percentage of CD107a^+^GZB^+^ cells. (C) Tr1- and Th0-cell lines were co-cultured with p815 cells pre-incubated with anti-CD226 mAb at 10:1 (E:T) ratio, and degranulation was determined by co-expression of CD107a and GZB in CD4^+^ T cells. One donor out of five donors performed in two independent experiments is shown. Numbers represent percentage of CD107a^+^GZB^+^ cells.

To dissect the molecular mechanism underlying the target specificity of Tr1 cells, we investigated adhesion and signaling molecules involved in Tr1-myeloid APC interaction. Both CD14^+^ and CD1c^+^ cells expressed higher levels of CD54 compared to T and B cells (not shown), and Tr1 cells expressed LFA-1 (CD18/CD11a), the CD54 ligand (Supporting Information Fig. 6). Addition of neutralizing anti-CD18 mAb blocked degranulation of Tr1-cell lines when co-cultured with CD14^+^ or CD1c^+^ cells (Fig. 6A). CD54 expression on target cells is specifically involved in the formation of a stable immunological synapse essential for cytotoxicity mediated by NK cells and CTLs 14. Therefore, our findings suggest that the high expression of CD54 on myeloid cells is responsible for the formation of a stable and prolong interaction with Tr1 cells, leading to lysis of the target cell.

We next investigated whether CD2, which is implicated not only in the adhesion between NK cells and its target cells, but also in NK-cell activation 15, contributes to Tr1-cell activation. Anti-CD2-bearing p815 cells promoted a strong Tr1-cell degranulation in the presence of GZB, whereas they induced degranulation of Th0 cells in the absence of GZB (Fig. 6B). Notably, CD58 is expressed at high levels in both CD14^+^ and CD1c^+^ cells (not shown) and Tr1 cells are CD2 positive (Supporting Information Fig. 6). These results demonstrate that activation of Tr1 cells via CD2 is required for cytotoxic activity mediated by Tr1 cells and suggest that CD58/CD2 interaction plays a key role in the killing of myeloid cells.

DNAM-1 (CD226) is an adhesion/signaling molecule that contributes to the NK-mediated lysis of DCs 16. Tr1-cell lines and Tr1-cell clones express high levels of CD226 (Supporting Information Fig. 6 and data not shown), and CD155 and CD112, the ligands of CD226, are specifically expressed on myeloid APC 17. We thus investigated whether CD226 contributed to Tr1-mediated lysis of myeloid cells. Anti-CD226-bearing p815 cells promoted Tr1-cell degranulation in the presence of GZB, whereas they did not induce degranulation of Th0 cells (Fig. 6C). Moreover, addition of anti-CD226 mAb significantly increased degranulation of Tr1 cells when co-cultured with CD14^+^ cells (21% versus 16% of CD107a^+^GZB^+^ cells in cultures in the presence or absence of anti-CD226, respectively, *p*=0.0016, Supporting Information Fig. 7). Similar results were obtained when CD1c^+^ cells were used as target cells (18% versus 15% of CD107a^+^GZB^+^ cells in cultures in the presence or absence of anti-CD226, respectively, *p*=0.02, Supporting Information Fig. 7). These results demonstrate that activation via CD226 is required for cytotoxicity mediated by Tr1 cells, and that specific interaction between CD155 and CD112 on myeloid cells and CD226 on Tr1 cells leads to Tr1-cell activation and degranulation.

### In vivo correlation between high percentages of GZB-expressing and IL-10-producing CD4^+^ T cells

We next investigated whether the relation between GZB expression and IL-10-producing Tr1 cells occurs also in vivo. We showed that IL-10 production and presence of Tr1 cells correlate with PMC in β-thal patients after HSCT (4 and data not shown). In peripheral blood of these PMC patients, the percentage of CD4^+^GZB^+^ T cells was higher compared to HD before and after stimulation (7.9% versus 3.9%, *p*=0.04 and 24% versus 3.5%, *p*=0.0001, respectively), and associated with higher frequencies of IL-10-producing CD4^+^ T cells 4 (Supporting Information Fig. 8A and B). Similar results were obtained by analyzing peripheral blood of β-thal patients tolerized after repetitive exposure to allo-Ag during multiple transfusions and prior-HSCT (Supporting Information Fig. 8C and D, and Fig. 9). Importantly, a correlation between the percentages of CD4^+^GZB^+^ T cells and the frequencies of IL-10-producing CD4^+^ T cells in PBMCs from both patients and HD was observed (Supporting Information Fig. 8E), suggesting that IL-10 modulates GZB expression also in vivo. Overall, these results indicate that in two different clinical conditions associated with tolerance, the presence of IL-10-producing cells correlates with GZB-expressing cells.

## Discussion

In this study, we define the cellular and molecular mechanisms underlying the cytotoxicity mediated by human Tr1 cells. We demonstrate that human Tr1 cells, in vitro generated and ex vivo isolated, express and release high levels of GZB, and specifically lyse target cells of myeloid origin, but not T and B lymphocytes. The mechanism of Tr1-mediated cytotoxicity is dependent on GZB and PRF, requires HLA class I recognition, LFA-1-mediated adhesion, and activation via KIRs, CD2, and CD226. GZB expression correlates with the amounts of IL-10 produced by Tr1 cells in vitro and in vivo, since high frequencies of IL-10-producing CD4^+^ T cells in tolerant β-thal patients are associated with elevated occurrence of CD4^+^GZB^+^ T cells. These results demonstrate that, in addition to suppression of effector T cells via cytokine secretion, expression of PD-1 and CTLA-4 18 and adenosine production 19, a key function of Tr1 cells is the GZB-dependent killing of myeloid cells.

Tr1-cell clones isolated from PMC β-thal patients express higher levels of GZB compared to Th0-cell clones 4. Furthermore, IL-10-producing Tregs generated by CD3/CD46 cross-linking, preferentially express GZB and lyse different target cells 10. In the present study, we show that IL-10-producing Tr1 cells, generated in vitro or ex vivo isolated, not only express, but also secrete GZB, which mediates the cytotoxic activity of Tr1 cells. Furthermore, we demonstrate for the first time that Tr1 cells specifically kill cells of myeloid origin.

Despite the fact that Tr1 cells are CD4^+^ T cells, they require recognition and activation via HLA class I molecules expressed on target cells to lyse myeloid cells, indicating that cytotoxicity mediated by Tr1 cells is Ag-independent. This mechanism of target recognition by Tr1 cells resembles the Ag-nonspecific-mediated recognition and activation of NK cells 20, and is opposed to the Ag-specific activation of CD8^+^ and CD4^+^ CTLs 21–23. However, in contrast to NK cells, which kill target cells lacking HLA class I molecules when regulation mediated by inhibitory receptors (i.e. KIRs) is missing, Tr1 cells kill target cells upon HLA class I molecule recognition and subsequent activation, indicating that Tr1-mediated cytotoxicity is triggered by activatory receptors recognizing HLA class I. In line with this finding, we showed that Tr1 cells express different activatory KIRs, which are probably involved in their activation and consequent lytic activity 24, 25.

The myeloid cell killing by Tr1 cells is attributable to the high levels of CD54, CD58, CD155, and CD112 on CD14^+^ and CD1c^+^ cells. CD54/LFA-1 and CD58/CD2 interactions not only contribute to a stable adhesion leading to the formation of lytic immunological synapse by NK cells 26, but also participate in their activation 27–29. Neutralizing mAb against CD18 efficiently abrogates the Tr1-mediated cytotoxicity, and activation of Tr1 cells through CD2 leads to GZB release independently from TCR engagement. Thus, despite the need for Tr1 cells to be activated via their TCR to secrete the immunomodulatory cytokines IL-10 and TGF-β, and exert their specific suppressive activity, once activated, Tr1 cells acquire GZB expression and lyse myeloid cells in an Ag-nonspecific CD2-mediated manner. This effect concurs with the activation of Tr1 cells via CD226-mediated signaling, and results in the polarized degranulation and release of both GZB and PRF, and in myeloid cell killing. CD226 is known to be critically involved in the NK-mediated killing of myeloid APCs, since CD155 and CD112, the ligands of CD226, are specifically expressed by monocytes and myeloid DCs 16, 17, 30–32. Since Tr1 cells express LFA-1, CD2, and CD226, we can speculate that their target specificity depends upon the array and density of expression of their ligands, CD54, CD58, and importantly, CD155 and CD112 on myeloid cells. The combination of signaling through these receptors, in association with activation by KIRs, is necessary to achieve the threshold required to properly activate Tr1-cell lytic activity. Moreover, our results showed that CD226 not only concurs with activation of Tr1 cells, but also confers their myeloid target cell specificity.

In the present study, we also demonstrated that the expression of GZB by Tr1-cell lines and cell clones correlates and is intrinsically associated with the presence of IL-10 in culture. The direct role of IL-10 in promoting and maintaining GZB expression in CD4^+^ T cells in vitro is supported by previous reports 13, 33 and by results obtained with PBL cultured in the presence of IL-10, which upregulated GZB (Serafini G., unpublished observations), and with human CD4^+^ T cells transduced with a lentiviral vector encoding for human IL-10, which constitutively express high levels of GZB (Andolfi G., unpublished observation). Here, we demonstrate that GZB expression by Tr1-cell clones is dependent on their autocrine IL-10 production. Tr1 cells are Ag-specific and produce IL-10 upon TCR stimulation 3. Thus, GZB expression by Tr1 cells is specific and occurs primarily when Tr1 cells are activated. This effect is independent of TGF-β and opposed to that observed in murine CD8^+^ CTLs 34. In line with these in vitro observations are in vivo results, where high expression of GZB is associated with circulating IL-10-producing T cells. Similarly, high frequency of IL-10-producing T cells 4 and CD4^+^GZB^+^ T cells is observed in tolerant patients (PMC and poly-transfused β-thal patients). Thus, these findings indicate that GZB is associated with IL-10 in vitro and in vivo, and can be used as surrogate marker for Tr1 cells in vivo.

Tr1 cells suppress T-cell responses mainly via IL-10 and TGF-β, secreted upon Ag-specific TCR activation. These immunomodulatory cytokines directly inhibit effector T-cell proliferation and expression of HLA class II and costimulatory molecules on APC, which indirectly suppress effector T cells 3. We now provide evidence that IL-10 produced by Tr1 cells upon TCR activation also directly induces GZB expression in Tr1 cells, which in turn acquire the ability to kill monocytes and myeloid DCs in an Ag-nonspecific manner. Based on these findings, we propose that selective depletion of myeloid APC by Tr1 cells, during an active immune response, represents an additional bystander mechanism of suppression that amplifies the tolerogenic loop induced by IL-10 and TGF-β produced by Tr1 cells.

## Materials and methods

Material and methods are reported in full as Supporting Information (available on the European Journal of Immunology website).

Human peripheral blood was obtained from HD upon informed consent in accordance with local ethical committee approval (TIGET PERIBLOOD) and with the World Medical Association's Helsinki Declaration.

### T-cell differentiation

Tr1- and Th0-cell lines were differentiated using murine L cells transfected with hCD32, hCD80, and hCD58 and supplemented with anti-CD3 mAb (100 ng/mL; OKT3, Jansen-Cilag, Raritan, NJ, USA) as previously described 5.

### Establishment of T-cell clones

T-cell clones were obtained from CD4^+^ cells by limiting dilution at 0.3 cells/well in the presence of a feeder cell mixture and soluble anti-CD3 mAb (1 μg/mL; OKT3, Jansen-Cilag) in X-VIVO 15 medium (BioWhittaker, Verviers, Belgium) supplemented with 5% pooled human AB serum (BioWhittaker), 100 IU/mL penicillin/streptomycin (BioWhittaker) as previously described 4.

### Cytotoxic assay

Cytotoxicity was assessed in a standard 4 h ^51^Cr release assay, as previously described 35. U937, K562, Daudi, and Jurkat target cell lines used were kindly provided by Dr. K. Fleischhauer. In some experiments, concanamycin A (CMA, Sigma-Aldrich, St Louis, MO, USA), Z-AAD-CMK (Calbiochem, San Diego, CA, USA), anti-HLA-I mAb (clone W/632, BioLegend), anti-HLA-G mAb (clone 87G, Exbio Praha, Nad Safinou, Czech Republic), and isotype controls (IgG2a,k, BD Pharmingen, San Diego, CA, USA) were added at the indicated concentrations.

### CD107a/GZB mobilization assay

T-cell degranulation was evaluated in a CD107a flow cytometric assay, according to a protocol adapted from Alter et al. 36. Briefly, 10^5^ cells from T cells were plated in IMDM (BioWhittaker) supplemented with 10% FCS (BioWhittaker), 100 IU/mL penicillin/streptomycin (BioWhittaker), 2 mM l-Glutamine (BioWhittaker), with anti-CD107a mAb (20 μL/mL; BD Pharmingen), in 96-well round-bottom plates, in the presence or absence of 10^4^ cell lines or freshly isolated target cells at 37°C. After 3 h, monensin A (Sigma-Aldrich) was added (30 μg/mL). After additional 3 h of incubation, cells were washed and stained with anti-CD4, and anti-GZB mAb. In some cultures anti-HLA-I mAb (20 μg/mL, clone W6/32, BioLegend), anti-HLA-G mAb (20 μg/mL, clone 87G, Exbio Praha), anti-CD18 mAb (25 μg/mL, clone TS1/18, BioLegend), anti-CD226 (10 μg/mL, clone 102511, R&D Systems, Minneapolis, MN, USA), and Ig isotype controls (IgG2a,k, and IgG1, respectively, BD Pharmingen) were added. 5×10^5^ p815 were incubated 30 min with 5 μg/mL anti-CD2 mAb (clone RPA-2.10, BD Pharmingen) or 10 μg/mL anti-CD226 (clone 102511, R&D Systems) and subsequently washed, before being used as target cells.

### Statistical analysis

Mean values were reported as Mean±SE. Mann–Whitney test was used to determine the statistical significance of the data. Two-tailed analysis was performed, unless not specified in the text. Significance was defined as ^*^p≤0.05; ^**^p≤0.005; and ^***^p≤0.0005. Statistic calculations were performed with the Prism program 5.0 (GraphPad Software).

## Abbreviations

GZA: granzyme A
GZB: granzyme B
HD: healthy donor
HSCT: hematopoietic stem cell transplantation
IONO: ionomycin
KIR: killer cell Ig-like receptors
LFA: lymphocyte function-associated antigen
PMC: persistent mixed chimerism
PRF: perforin
SFU: spot forming units
Tr1: type 1 regulatory T
β-thal: β-thalassemic
