# Supplementary material for: Killing of myeloid APCs via HLA class I, CD2 and CD226 defines a novel mechanism of suppression by human Tr1 cells
Source: Eur J Immunol. 2011 Apr 6;41(6):1652–62. doi: 10.1002/eji.201041120 (PMC3116154; doi:10.1002/eji.201041120)
Supplement: Supplementary file 1 [file eji0041-1652-SD1.pdf]

European Journal of Immunology

Supporting Information

for

DOI 10.1002/eji.201041120

Killing of myeloid APCs via HLA class I, CD2 and CD226 defines a novel mechanism of suppression by human Tr1 cells

Chiara F. Magnani, Giada Alberigo, Rosa Bacchetta, Giorgia Serafini,
Marco Andreani, Maria Grazia Roncarolo
and Silvia Gregori

© 2011 WILEY-VCH Verlag GmbH & Co. KGaA, Weinheim

Supplementary Information

Killing of myeloid APC *via* HLA Class I, CD2 and CD226 defines a novel mechanism of suppression by human Tr1 cells

Chiara F. Magnani, Giada Alberigo, Rosa Bacchetta, Giorgia Serafini, Marco Andreani, Maria Grazia Roncarolo, and Silvia Gregori

Supplementary Materials and Methods

Cell isolation and purification

PBMC were isolated by centrifugation over Lymphoprep Ficoll gradients (Fresenius Kabi Norge AS, Halden, Norway). $CD4^+$ T lymphocytes were purified using the untouched $CD4^+$ T Cell Isolation Kit II (Miltenyi Biotec, Auburn, CA) according to manufacture's instructions. Naïve $CD4^+CD45RO^-$ T lymphocytes were purified by CD45RO MicroBeads (Miltenyi Biotec). NK cells were purified by negative Dynabeads selection for $CD3^+$, $CD19^+$, and $CD14^+$ cells (Dynal, Oxoid, Italy), as previously described [1]. $CD14^+$ and $CD1c^+$ cells were purified using CD14 MicroBeads (Miltenyi Biotec) or using CD1c (BDCA-1) Dendritic Cell Isolation Kit (Miltenyi Biotec), respectively, according to manufacture's instructions. $CD3^+$ were enriched by negative Dynabeads selection for $CD19^+$, and $CD14^+$ cells and $CD19^+$ cells by positive Dynabeads selection for $CD19^+$ (Dynal, Oxoid, Italy) according to manufacture's instructions. IL-10-producing T cells were purified from polarized Tr1 cell lines stimulated for 4 h with immobilized anti-CD3 mAb (10 μg/mL; Jansen-Cilag, Raritan, NJ, USA) and PMA (10 ng/ml; Sigma, St Louis, MO, USA) or PBMC activated with CytoStim (Miltenyi Biotec) for 16 h, using the IL-10-secretion assay (Miltenyi Biotec), according to the manufacturer's instruction.

Patients

Nineteen pts affected of β-thalassemia with age ranged from 2 to 17 y have been transplanted from HLA-identical sibling donors at the San Raffaele Scientific Institute since 2005 and at the Istituto Mediterraneo IME since 2004. Before transplantation, the pts were subjected to not-leucodepleted multiple blood transfusions to overcome the inefficient synthesis of hemoglobin. We analyzed ten pts who remained in a state of PMC, in which patient and donor cells co-exist for longer than 2 y after HSCT. The study was approved by the Ethical Committee of the Policlinico Tor Vergata, Rome and by the Ethical Committee of San Raffaele Scientific Institute, Milan. Informed consent from pts was obtained according to institutional guidelines and to the Helsinki Declaration.

Cytokine detection

10^6 cells/ml were stimulated with immobilized anti-CD3 mAb (10 μg/mL; Jansen-Cilag) and soluble anti-CD28 mAb (1 μg/mL; BD Pharmingen). After 24 (IL-2), and 48 h (other cytokines), culture supernatants were harvested and levels of cytokines were determined by ELISA according to the manufacturer's instruction (BD Biosciences). The limits of detection were: IFN-γ: 60 pg/ml; IL-10: 19 pg/ml; IL-4: 9 pg/ml; IL-2: 15 pg/ml; IL-17: 30 pg/ml.

Flow cytometry analysis

T cells were tested for the expression of GZB (Caltag MedSystem, Buckingham, UK), GZA (BD Pharmingen), PRF (Biolegend), IL-10 (BD Pharmingen), IL-4 (BD Pharmingen), CD2 (BD Pharmingen), LFA-1 (BD Pharmingen), and CD226 (Biolegend). For intracytoplasmic staining, T cells were stained with anti-CD4 mAb (BD Pharmingen) before fixation, permeabilization (Fixation/Permeabilization Solution Kit, BD Bioscience, San Diego, CA, USA) and incubation with mAb. Samples were acquired using a BD FACS Canto flow cytometer (BD Biosciences), and data were analyzed with FCS express (De Novo Software, Los Angeles, CA). Quadrant markers were set accordingly to unstained controls.

ELISPOT assay

GZB-releasing T cells were evaluated by GZB ELISPOT kit (eBiosciences, San Diego, CA, USA) according to the manufacturer's instruction. Briefly, T cells were plated at the starting concentration of $0.5x10^6$ T cells/ml in 200 µl of RPMI (BioWhittaker, Verviers, Belgium) supplemented with 10% FCS (BioWhittaker), 100 IU/ml penicillin/streptomycin (BioWhittaker), 2 mM L-Glutamine (BioWhittaker). The test was performed in duplicate for 6 consequence dilutions 1:2 and incubated for 48 h. The spots were counted by KS ELISPOT system (Zeiss Vision, Göttingen, Germany).

Apoptosis detection

Target viability was measured by Annexin V and 7-ADD (BD Pharmingen, San Diego, CA, USA) staining, according to manufacture's protocols, gating on target cells previously labeled with 5-(and-6)-carboxy fluorescein diacetate succinimidyl ester (CFSE, 1 μM, Molecular Probes, Eugene, OR).

Expression profile of KIR genes

Total RNA was extracted with RNeasy Mini kit (Qiagen, Hilden, Germany) from T cells, and cDNA was synthesized with high-capacity cDNA archive kit (Applied Biosystems, Foster City, CA), according to manufacture's instructions. Detection of human KIR genes at the level of mRNA were performed by the KIR typing kit (Miltenyi Biotec), according to manufacture's instructions.

References

1 **Draghi, M., Yawata, N., Gleimer, M., Yawata, M., Valiante, N. M. and Parham, P.,** Single-cell analysis of the human NK cell response to missing self and its inhibition by HLA class I. *Blood* 2005. **105:** 2028-2035.

Supplementary Figure 1

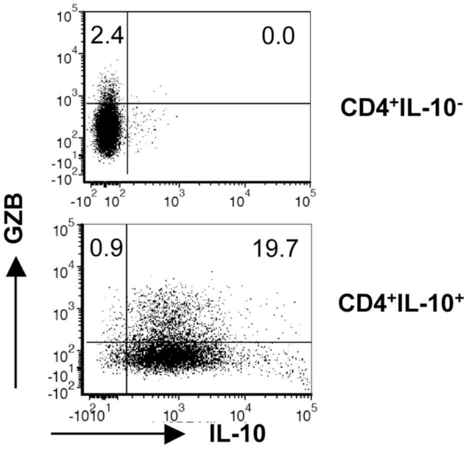

Figure S1. Circulating IL-10-producing CD4$^+$ T cells express GZB. PBMC from HD were activated with CytoStim (Miltenyi Biotec) for 16 h and IL-10-producing cells were purified using the IL-10-secretion assay (Miltenyi Biotec). IL-10 and GZB expression was determined in CD4$^+$IL-10$^+$ and CD4$^+$IL-10$^-$ T cells by intracytoplasmic staining. One donor out of 2 donors tested is shown. Numbers represent percentage of positive cells.

Supplementary Figure 2

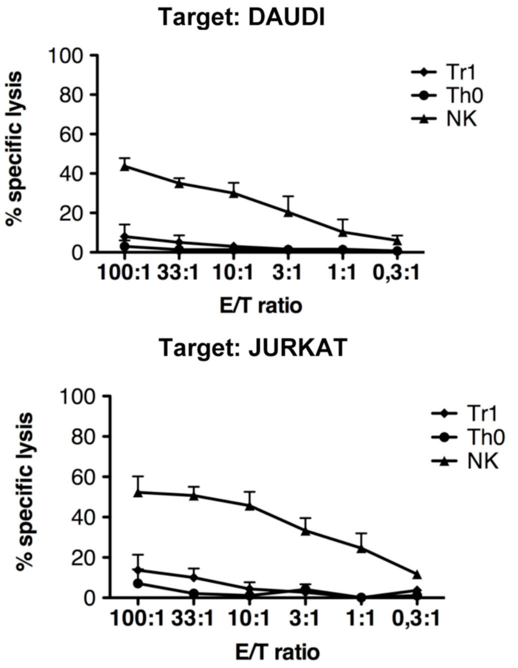

Figure S2. Tr1 cell lines do not lyse Daudi and Jurkat cells. Cytotoxic activity of Tr1 and Th0 cell lines against Daudi and Jurkat target cell lines was determined by ^{51}Cr release standard assay. As positive control NK cell lines from the same HD were used. Mean ± SE of 3 independent donors for Daudi and 4 for Jurkat performed in duplicate are reported.

Supplementary Figure 3

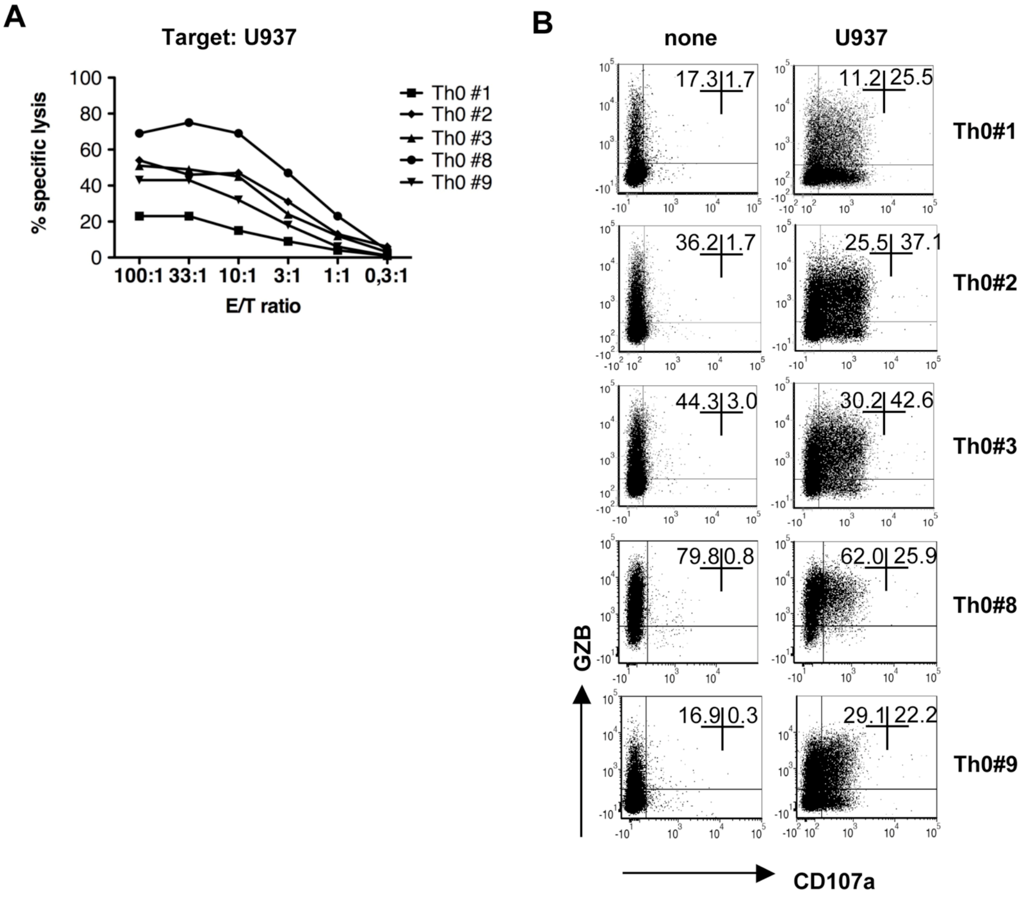

Figure S3. Th0 cell clones lyse target cell of monocytic origin regardless IL-10- and GZB-expression. (A) Cytotoxic activity of 5 Th0 cell clones against U937 target cell line was determined by ^{51}Cr release. (B) In parallel, Th0 cell clones were co-cultured with U937 target cell line at 10:1 (E:T) ratio and cytotoxicity was measured by co-expression of CD107a and GZB in CD4^{+} T cells. Numbers represent percentage of CD107a^{+}GZB^{+} cells.

Supplementary Figure 4

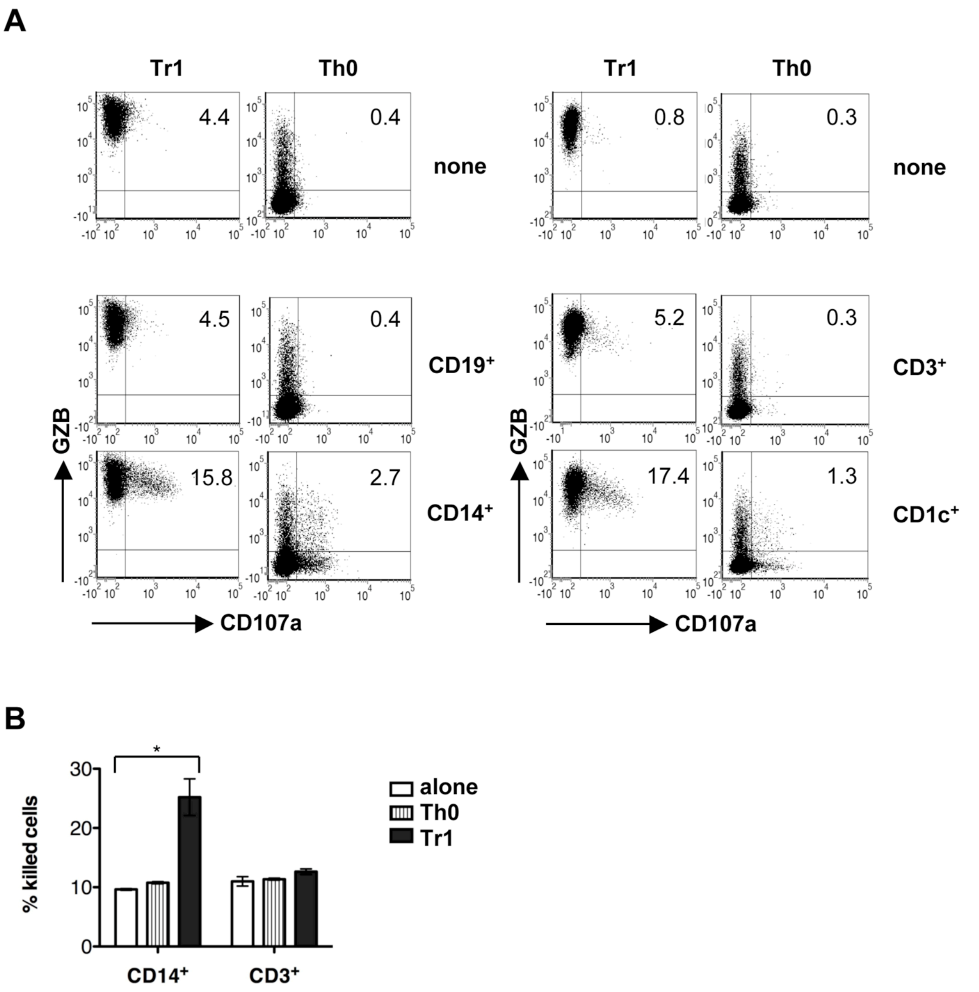

Figure S4. Tr1 cell lines specifically lyse primary monocytes and myeloid DC. (A) Tr1 and Th0 cell lines were co-cultured with autologous freshly isolated CD19$^+$, CD14$^+$, CD3$^+$, and CD1c$^+$ cells at 10:1 (E:T) ratio, and cytotoxic activity was measured by co-expression of CD107a and GZB in CD4$^+$ T cells. One representative donor out of 3 (CD19$^+$), 3 (CD14$^+$), 4 (CD3$^+$), and 3 (CD1c$^+$) performed in 2 independent experiments is shown. Numbers represent percentage of CD107a$^+$GZB$^+$ cells. (B) Freshly isolated allogeneic CD14$^+$, and CD3$^+$ T cells stained with CSFE were left alone or co-cultured with Tr1 and Th0 cell lines at 10:2 (E:T) ratio. After culture, cells were labeled with PE-conjugated Annexin V and 7-AAD (BD Pharmingen), and the percentages of killed cells were determined as AnnexinV$^+$7-AAD$^+$ plus AnnexinV$^-$7-AAD$^+$ in CSFE$^+$ target cells. Mean±SE of 5 donors performed in 2 independent experiments is shown. *P≤0.05.

Supplementary Figure 5

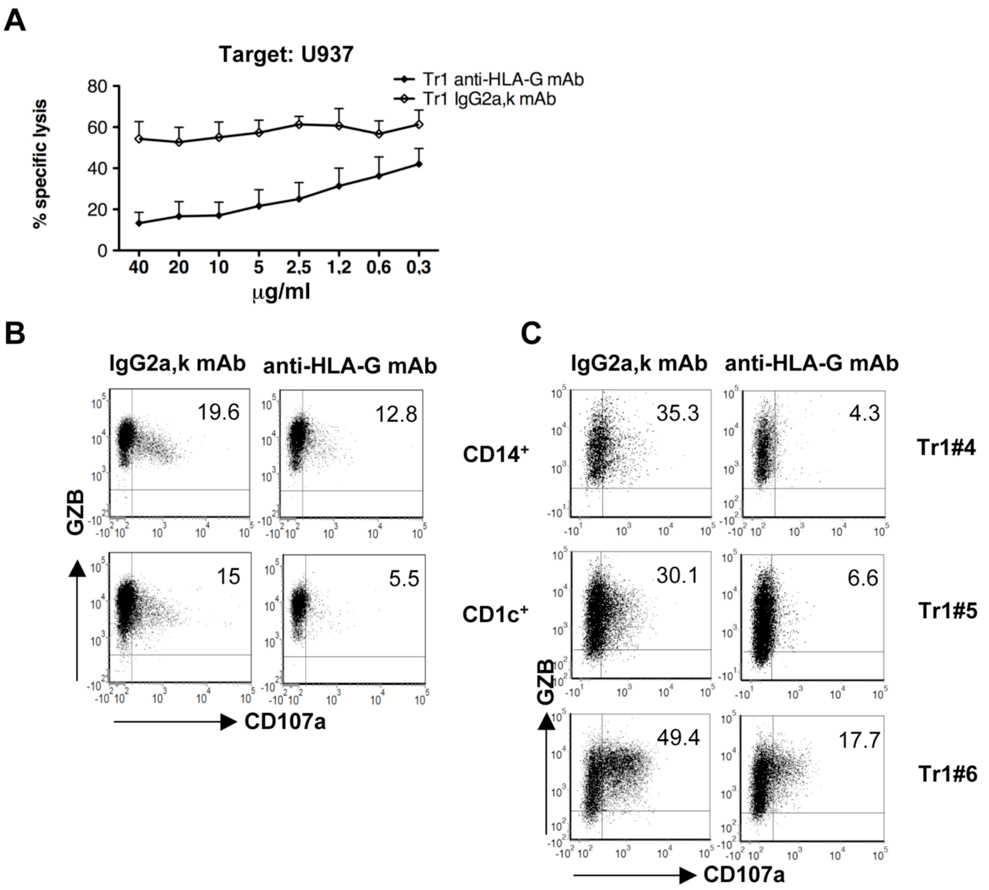

Figure S5. Cytotoxicity mediated by Tr1 cells is partially HLA-G-dependent. (A) Tr1 cell lines were co-cultured with U937 target cell line at 100:1 (E:T) ratio in presence of anti-HLA-G mAb or IgG2a,k isotype control mAb at the indicated concentrations, and cytotoxic activity was determined by ^{51}Cr release. Mean ± SE of 3 donors performed in 2 independent experiments are reported. (B) Tr1 cell lines were co-cultured with freshly isolated autologous CD14$^+$ and CD1c$^+$ cells at 10:1 (E:T) ratio in the presence of anti-HLA-G mAb or IgG2a,k isotype control, and degranulation in the presence of GZB was measured by co-expression of CD107a and GZB in CD4$^+$ T cells. One representative donor out of 3 donors performed in a single experiment is shown. Numbers represent percentage of CD107a$^+$GZB$^+$ cells. (C) Tr1 cell clones were co-cultured with U937 target cell lines at 10:1 (E:T) ratio in the presence of anti-HLA-G mAb or IgG2a,k isotype control (20 µg/ml), and degranulation was measured by co-expression of CD107a and GZB in CD4$^+$ T cells. Numbers represent percentage of CD107a$^+$GZB$^+$ cells.

Supplementary Figure 6

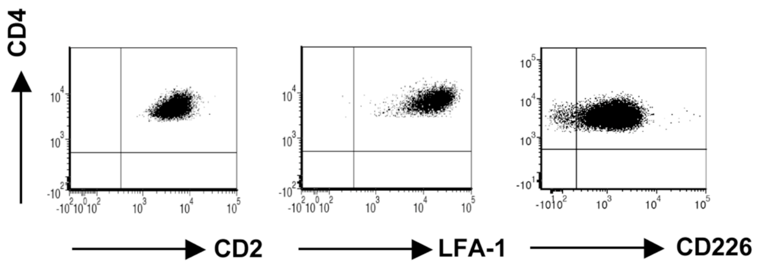

Figure S6. Tr1 cells express CD2, LFA-1, and CD226. CD2, LFA-1 and CD226 expression was determined in Tr1 T cell lines. One donor out of 5 donors tested is shown.

Supplementary Figure 7

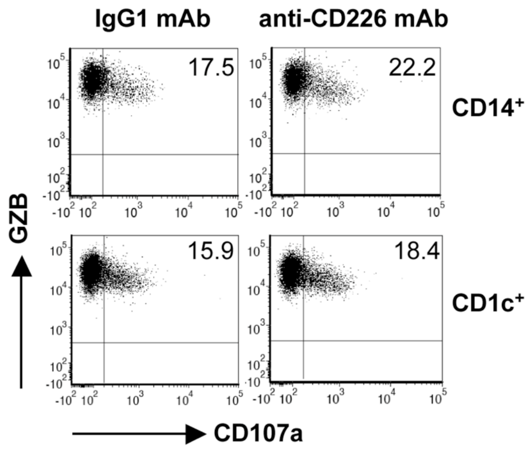

Figure S7. Tr1-mediated cytotoxicity requires activation *via* CD226. Tr1 cell lines were co-cultured with $CD14^+$ or CD1c cells at 10:1 (E:T) ratio in the presence of anti-CD226 mAb or IgG1 isotype control, and degranulation in the presence of GZB was measured by co-expression of CD107a and GZB in $CD4^+$ T cells. One donor out of 6 donors for $CD14^+$ cells and of 4 donors for $CD1c^+$ cells tested in 3 independent experiments is shown. Numbers represent percentage of $CD107a^+GZB^+$ cells.

Supplementary Figure 8

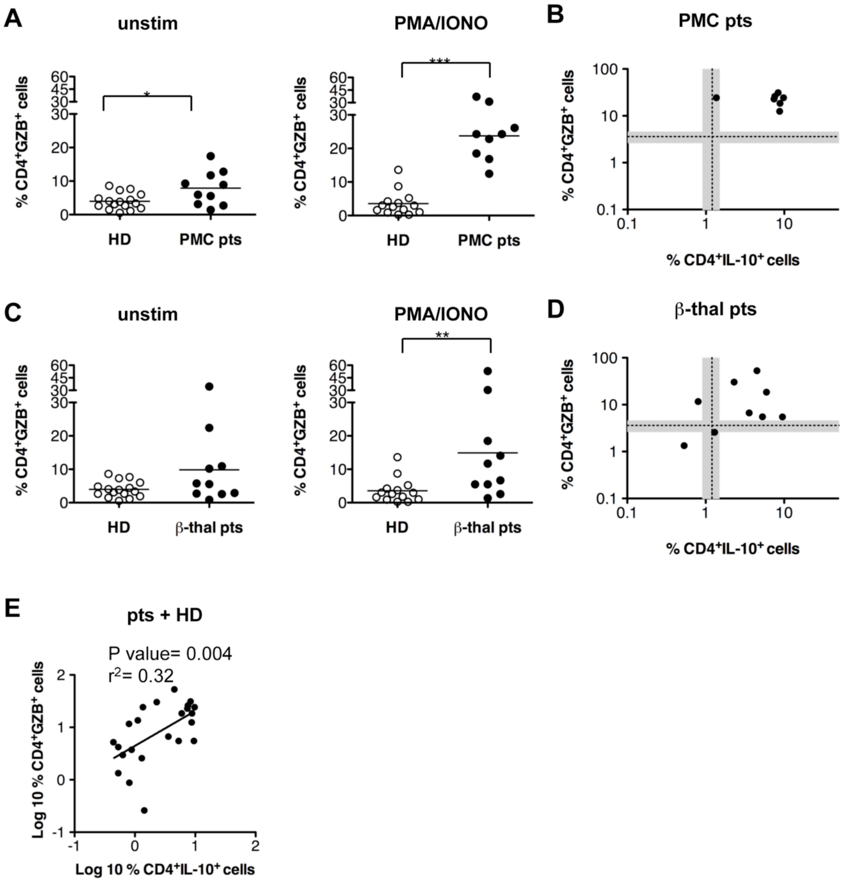

Figure S8. High frequencies of GZB-expressing CD4$^+$ T cells correlated with elevated percentages of IL-10-producing CD4$^+$ T cells *in vivo*. PBMC from PMC pts, β-thalassemic pts prior HSCT and HD were activated with PMA (10 ng/ml; Sigma) and IONO (150 ng/mL; Sigma) or left unstimulated for 12 h in the presence of brefeldin A (10μg/ml; Sigma). (A) Percentages of CD4$^+$GZB$^+$ T cells in each of 10 PMC pts and 16 HD tested for unstimulated condition and in each of 9 PMC pts and 14 HD tested for PMA/IONO are presented. Black lines represent the mean percentages of CD4$^+$GZB$^+$ T cells. (B) Plot represents percentages of CD4$^+$IL-10$^+$ T cells vs. percentages of CD4$^+$GZB$^+$ T cells in each of 7 PMC pts tested after activation with PMA/IONO. Dashed lines represent the mean percentages of CD4$^+$IL-10$^+$ T cells of 12 HD and CD4$^+$GZB$^+$ T cells of 14 HD and the grey bar ± SE. (C) Percentages of CD4$^+$GZB$^+$ T cells in each of 10 β-thalassemic pts prior HSCT and 16 HD tested for unstimulated condition and in each of 10 β-thalassemic pts and 14 HD tested for PMA/IONO are presented. Black lines represent the mean percentages of CD4$^+$GZB$^+$ T cells. (D) Plot represents percentages of CD4$^+$IL-10$^+$ T cells vs. percentages of CD4$^+$GZB$^+$ T cells in each of 9 β-thalassemic pts prior HSCT tested after activation with PMA/IONO. Dashed lines represent the mean percentages of CD4$^+$IL-10$^+$ T cells of 12 HD and CD4$^+$GZB$^+$ T cells of 14 HD and the grey bar ± SE. (E) Plot represents percentages of CD4$^+$IL-10$^+$ T cells vs. percentages of CD4$^+$GZB$^+$ T cells in each of 7 PMC pts, 9 β-thalassemic pts prior HSCT, and 7 HD tested after activation with PMA/IONO. The P value of the correlation and the coefficient of determination (r^2) are reported (two-tailed test). * $P \leq 0.05$, ** $P \leq 0.005$, and *** $P \leq 0.0005$ (two-tailed test)

Supplementary Figure 9

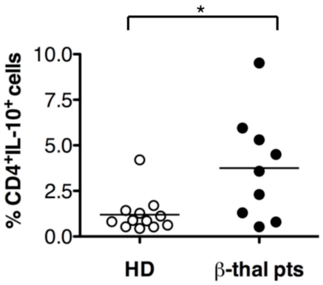

Figure S9. High percentage of CD4$^+$IL-10$^+$ T cells in β-thalassemic pts prior-HSCT. PBMC from β-thalassemic pts prior HSCT and HD were activated with PMA (10 ng/ml; Sigma) and IONO (150 ng/mL; Sigma) for 12 h in the presence of brefeldin A (10μg/ml; Sigma). Percentages of CD4$^+$IL-10$^+$ T cells in each of 9 β-thalassemic pts and 12 HD tested after activation with PMA/IONO are presented. Black lines represent the mean percentages of CD4$^+$IL-10$^+$ T cells. * $P \leq 0.05$, (two-tailed test).
